# Catch-Up Growth in Infants and Young Children With Faltering Growth: Expert Opinion to Guide General Clinicians

**DOI:** 10.1097/MPG.0000000000003784

**Published:** 2023-03-28

**Authors:** Richard Cooke, Olivier Goulet, Koen Huysentruyt, Koen Joosten, Anuradha Vaman Khadilkar, Meng Mao, Rosan Meyer, Andrew M. Prentice, Atul Singhal

**Affiliations:** From * University of Tennessee, Knoxville, TN; † Université Paris Descartes, Paris, France; ‡ Vrije Universiteit Brussel, Brussels, Belgium; § Erasmus MC-Sophia Children’s Hospital, Department of Pediatric & Neonatal Intensive Care, Rotterdam, Netherlands; ∥ Hirabai Cowasji Jehangir Medical Research Institute, Pune, India; ¶ Sichuan University, Chengdu, China; # Imperial College, London, United Kingdom; ** Winchester University, Winchester, United Kingdom; the †† Medical Research Council Unit @ LSHTM, Banjul, Gambia; the ‡‡ Childhood Nutrition Research Centre, UCL Great Ormond Street Institute of Child Health, London, United Kingdom.

**Keywords:** accelerated growth, catch-up growth, faltering growth, growth

## Abstract

Faltering growth (FG) is a problem regularly seen by clinicians in infants and young children (<2 years of age). It can occur due to non-disease-related and disease-related causes and is associated with a wide range of adverse outcomes, including shorter-term effects such as impaired immune responses and increased length of hospital stay, and longer-term consequences, including an impact on schooling and cognitive achievements, short stature, and socioeconomic outcomes. It is essential to detect FG, address underlying causes and support catch-up growth where this is indicated. However, anecdotal reports suggest misplaced fear of promoting accelerated (too rapid) growth may deter some clinicians from adequately addressing FG.

An invited international group of experts in pediatric nutrition and growth reviewed the available evidence and guidelines on FG resulting from disease-related and non-disease-related effects on nutritional status in healthy term and small for gestational age infants and children up to the age of 2 years in low-, middle-, and high-income countries. Using a modified Delphi process, we developed practical consensus recommendations to provide clarity and practical recommendations for general clinicians on how FG should be defined in different young child populations at risk, how FG should be assessed and managed, and the role of catch-up growth after a period of FG. We also suggested areas where further research is needed to answer remaining questions on this important issue.

## Graphical abstract

**Figure:**
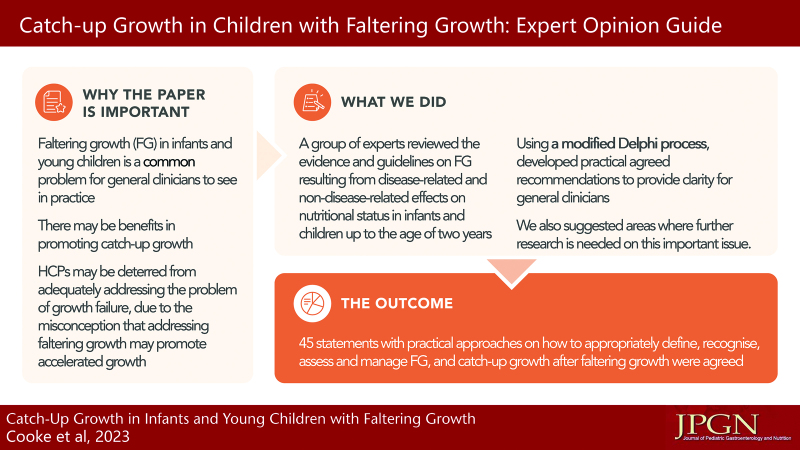

**What Is Known**

- Faltering growth (FG) in infants and young children (<2 years of age) is a common problem for general clinicians to see in clinical practice, especially in low-income settings.
- FG is associated with a range of adverse outcomes and there may be benefits in promoting *catch-up growth* where this is indicated.
- Health care professionals may be deterred from adequately addressing the problem of growth failure, due to the reported misconception that addressing FG may promote accelerated growth.

**What Is New**

- Recommendations are proposed to provide clinicians with practical approaches on how to appropriately recognize, assess, and manage FG in low-, middle-, and high-income countries. These are based on the available evidence and expert opinion where evidence is lacking.
- Recommendations for further research have been suggested.

## GROWTH AND FALTERING GROWTH (FG)

### Growth

Growth reflects a complex interaction between nutritional, genetic, hormonal, and environmental factors. It is “programmed” to occur within a “critical time frame” or “epoch” which if missed may not be recoverable (1–4). This means that even “short-term” deprivation during a “critical epoch,” for example, infancy, may be paralleled by long-term effects on organ growth and function (1–3).

As a “mirror of health,” the measurement of growth is an important and noninvasive tool during infancy and childhood (5), reflecting not only the health and nutritional status of an infant but also the quality of life of a population (6). Yet, what is or is not normal growth is not always clear. Normal is what happens frequently but does not imply healthiness; values outside the confidence range do not necessarily imply poor health. Nevertheless, growth generally “tracks” within 1 centile space (7).

### Faltering Growth

There are many definitions of FG (also referred to by other terms including growth faltering, “failure to thrive,” stunting, and growth failure). For example, the National Institute for Health and Care Excellence, which advises the National Health Service in the UK, suggested the following thresholds for concern about FG in infants: a fall across 1 or more weight centile spaces (1 centile space = 0.67 *z* scores) if birthweight was below the 9th centile; a fall across 2 or more weight centile spaces if birthweight was between the 9th and 91st centiles; a fall across 3 or more weight centile spaces if birthweight was above the 91st centile; when current weight is below the 2nd centile for age, whatever the birthweight (8).

Given the international nature of this review, the World Health Organization (WHO) definition of growth faltering seems most relevant to provide a simple, pragmatic approach for clinicians: a fall in weight for age (WFA) *z* score of ≥1.0 merits evaluation (6). Current definitions lack a timeframe for the period of growth faltering. We suggested that it was important to include a time frame for falling weight that should be sufficient to ensure that acute weight loss, such as that occurring rapidly with an episode of vomiting and diarrhea is not classified as growth faltering. Growth faltering also excludes the first 2 weeks after birth, when weight loss is expected (8).

Growth faltering potentially has an immediate effect on a child’s health and can be a marker for something serious, so it is clearly important that clinicians recognize growth faltering and manage it appropriately.

### Catch-Up Growth

**FIGURE 1.**
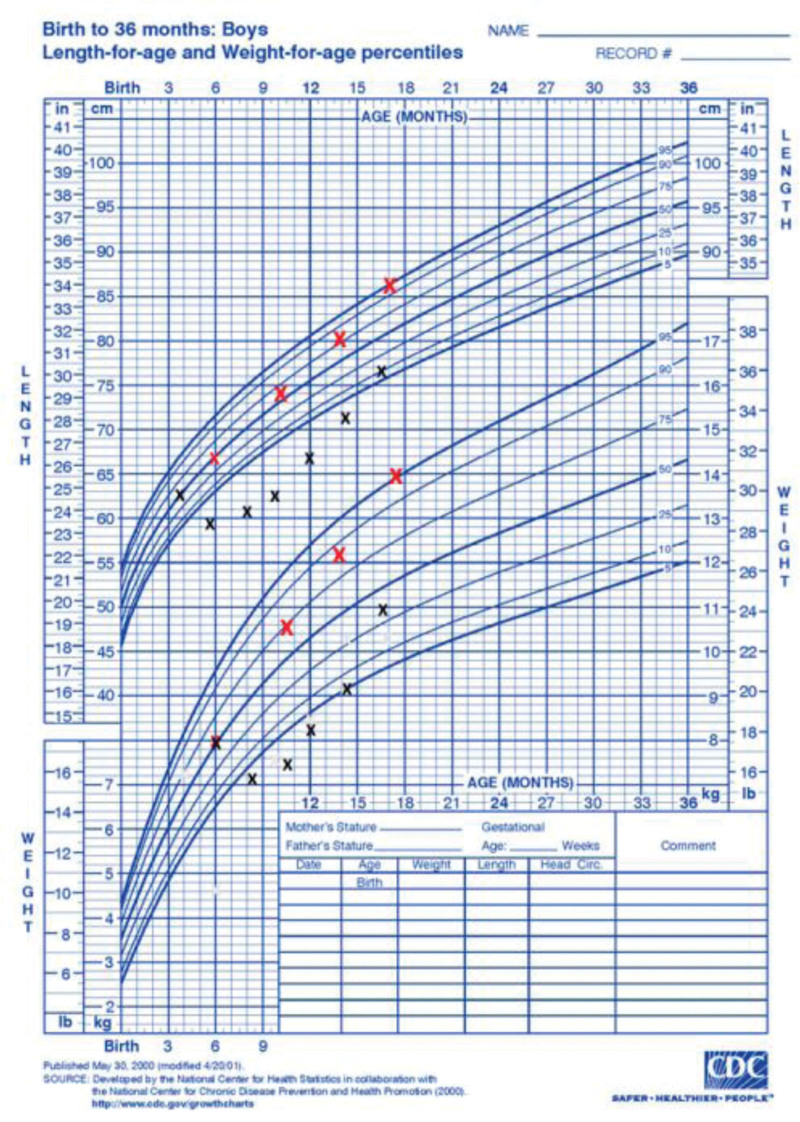
Graph illustrating the difference between catch-up growth and accelerated growth. x, catch-up growth; x, accelerated growth.

Catch-up growth was defined by Prader in 1963 as the “acceleration in growth *in response to recovery from illness or starvation*” (9,10). It has been defined as: a physiologic increase in WFA *z* score after a period of “growth faltering,” ideally to the original WFA *z* score (7) (Figure 1). de Wit et al (11) suggested that the term catch-up growth is mostly used for height, being defined as height velocity above normal following a transient period of growth inhibition.

The target for when to stop interventions designed to achieve catch-up growth (eg, increasing protein/energy intake) is currently poorly defined. Such a target would be helpful for clinical practice, to help prevent excessive weight gain. This can occur, for example, where parents, anxious about the weight of their child, consider “bigger, is better” and so continue to use high nutrient feeds after catch-up growth has been achieved.

We suggested that a pragmatic target for catch-up growth would be return to the child’s original centile or *z* score before experiencing growth faltering, given the child was in good nutritional status before the insult occurred. Acknowledging the importance of body composition, it was also suggested that attention should be paid to changes in length for age *z* score as a simple-to-measure approximate proxy of lean mass (12) Concerning body composition, weight for length and body mass index are widely used as early adiposity screening tools, but these parameters do not distinguish between fat mass (FM) and fat-free mass (FFM). There are a number of published anthropometry-based prediction equations for body composition in children in which skinfold thickness measurements were also taken into account (13–16). A further suggestion was to measure the ratio of brachial to head circumference as part of assessing malnutrition and recovery. Finally, it was acknowledged that in addition to WFA *z* score, mid-upper arm circumference is often used as a simple screening tool to assess growth and nutritional status in low- and middle-income countries (LMICs).

### The Difference Between Catch-Up Growth and Growth Acceleration

Growth acceleration is defined as upward-centile crossing for WFA and, in contrast to catch-up growth, is not in response to FG (17). It occurs spontaneously [eg, in small for gestational age (SGA) babies] and can be promoted, for example, by overfeeding or formula-feeding compared to breast-feeding. One proposed definition is an increase in WFA *z* score of ≥1.0 (7). Both catch-up growth and growth acceleration involve upward centile crossing in weight, but only catch-up growth follows recovery from illness or a period of under-nutrition (Figure 1).

This distinction between faster growth as a result of recovery catch-up growth or growth acceleration has different consequences for health outcomes. In contrast to the generally accepted short-term and long-term health benefits of promoting catch-up growth following growth faltering, growth acceleration for weight is associated with adverse effects on long-term health (18). For example, upward centile crossing for weight in infancy has been associated with later obesity in 45 of 46 studies (summarized in 6 systematic reviews (18–20) including an individual-level meta-analysis in 47,661 participants from 10 cohorts (20)). These associations are seen in both high-income and low-income countries (including South Africa, Mexico, Brazil, China, Sri Lanka, and India), for both weight gain and linear growth, in infants born preterm or at term, in infants with normal or low birthweight for gestation, and in both breast-fed and formula-fed infants (18–20). The effect size is large, with relative risk of obesity associated with faster infant weight gain ranging from 1.2 to as high as 5.7 (as reviewed in (18)).

Appropriate diagnosis, treatment, and prevention of growth faltering is widely accepted as best practice in pediatrics, but, anecdotally, there is a concern among some health care professionals that promotion of catch-up growth could have adverse consequences for long-term health because they are worried about the adverse health effects of rapid weight gain or growth acceleration (21). It is important to understand that catch-up growth and accelerated growth are different phenomena, with different definitions and associated with different health outcomes. Clinicians should address, identify, and manage FG appropriately and understand that this will not cause growth acceleration and excessive weight gain. Unhealthy weight gain in infants should be addressed as a separate issue, where clinicians should give parents appropriate advice and ensure they are not overfeeding their children. No clinician should let a child continue to experience FG because of misplaced fears about potentially inducing accelerated growth.

### Agreed Statements: Proposed Definitions of FG, Catch-Up Growth, Growth Acceleration, and Normal Growth (After an Insult)

**Growth faltering** is a fall in WFA *z* score of ≥1.0 that occurs over a period of 1 month or more and does not include the first 2 weeks after birth (6,8).

**Catch up growth** is increased growth velocity following recovery from illness or starvation. It is a physiologic increase in WFA *z* score after a period of “growth faltering,” ideally to original WFA *z* score.

**Accelerated growth/rapid growth** is upward crossing of centiles in weight (eg, an increase in WFA *z* score of ≥1.0) that is not preceded by growth faltering (6). It can occur both spontaneously (eg, in infants born SGA) and can be promoted (eg, as a consequence of overfeeding or formula-feeding compared to breast-feeding).

**Normal growth** is achieved once the child has “caught-up” and returned to the WFA *z* score or centile on which a child was growing before growth faltered.

## FG IN HIGH- AND MIDDLE-INCOME SETTINGS

Infants are particularly vulnerable to malnutrition because of their limited body reserves and their higher nutrient requirements for growth and development (22–24). Based on its etiology, FG can be (25):

- Illness-related, with or without infection/inflammation
- Non-illness-related, caused by environmental/behavioral factors associated with decreased nutrient intake/delivery (or both); this may be compounded by infection.

The etiology of growth faltering is often multifactorial and there may be interplay between illness and non-illness related FG. It may not be possible to identify a clear single cause.

## DISEASE-RELATED FG

Failure to thrive or FG may be the presenting symptom in a wide range of diseases, so inadequate weight gain and/or decreasing growth velocity for age should be considered as an early disease symptom in an otherwise apparently healthy child (26). The mechanisms of nutrient imbalance in disease-related FG include:

1. Reduced or restricted intake, due to sucking and swallowing disorders; insufficient intake from secondary anorexia (eg, cardiac and/or lung diseases, cancer, cerebral palsy); eating disorders.
2. Excessive losses, due to vomiting; intestinal malabsorption (eg, untreated celiac disease, cystic fibrosis, cholestasis, or intestinal failure including short bowel syndrome, intractable diarrhea, chronic intestinal pseudo-obstruction); protein-losing enteropathy (eg, intestinal lymphangiectasia, inflammatory bowel disease; severe dermatologic disease).
3. Increased requirements due to infection and inflammation (eg, pulmonary, cardiac, renal, hemato-oncologic, neurologic, endocrine diseases).

Static data (only 1 measurement of weight and height) is available on acute and chronic malnutrition in children admitted to hospital but there is no definitive data on FG, which requires dynamic data, at least 2 or 3 previous growth measurements over time. In reality, disease-related FG is likely to have started some time before hospital admission in most cases.

Current studies report very different rates of disease-related malnutrition according to hospital type and size, methodology, and the population selection. Illness-related malnutrition prevalence ranging from 6% to 51% has been reported in hospitalized children (25). The prevalence of malnutrition varies depending on the underlying medical or surgical conditions, with the highest prevalence in patients with multiple diagnoses and neurologic diseases (up to 44%). Disease-related FG/malnutrition also occurs frequently in patients with infectious diseases, cystic fibrosis, cardiovascular disease, oncologic diseases, and with gastrointestinal diseases (25). Critically ill children are at a particularly high risk of developing malnutrition and studies have shown that 14%–32% of infants admitted to pediatric intensive care units (PICU) have acute or chronic malnourishment on admission (27). It is important that pediatricians and nutritionists are alert to the risk of malnutrition and assess and monitor malnutrition as an important prognostic factor, in children admitted to hospital. Identifying those at risk is essential and could be helped by using validated nutritional risk scores/tools (eg, PYMS, STAMP, or STRONGkids) (28–30).

Disease-related FG can have serious short- and long-term consequences. Critically ill children who become malnourished during PICU stay show increased mortality, length of mechanical ventilation, and length of stay (31,32). Malnourished children also have increased rates of infection and longer-term behavioral problems, including impaired communication skills, attention-deficit, and hyperactivity disorders (33). However, most data on the adverse effects of malnutrition come from studies carried out in low-income countries, with very limited data on the long-term effects of FG in children in Western countries, although there is some evidence in children with congenital heart disease.

Considering the long-term consequences of FG, investigators relying on population- and community-based samples have found that most children with a history of FG have experienced growth recovery by school age (34–36). Although many continue to be shorter than age-matched peers they rarely experience growth deficits indicative of severe malnutrition. However, FG may have a small, though potentially important, impact on children’s cognitive performance. A meta-analysis in 2004 suggested that FG in infants may result in long-term problems in cognitive development with a 4.2 intelligence quotient point decrement (37,38). In another study, young children who had failure to thrive followed for up to 8 years had measurable intelligence quotient deficits as well as learning and behavioral difficulties (39). Chronic illness leading to stunting may have consequences on long-term growth and final size/target size and if FG caused by disease is not corrected it can develop into non-disease-related FG.

Nutritional support given to critically ill children with disease-related FG should take account of the phase (acute, stable and recovery) of critical illness (40). During the acute phase there is a considerable risk of overfeeding which can harm the patient and nutrient restriction might be beneficial in the early acute catabolic phase. During the stable and recovery phase, there will be a shift from catabolism to anabolism and nutritional support should focus on increasing high-quality protein and energy intake to enable tissue repair, recovery of organ function, growth and catch-up growth. Refeeding syndrome (RFS) may occur in case of management of a malnourished child especially if protein energy malnutrition is severe. RFS may be observed whatever the route of nutritional support (41–43)

To treat infants with FG a 3-step approach may be used: (1) normalize intake, (2) enrichment with protein/lipid/carbohydrate modules, and (3) protein-energy enriched (PEE) formula. So far, only a limited number of studies have focused on weight achievement when using PEE formulas in children that are non-critically ill or critically ill (44–46).

### Agreed Statements: Disease-Related FG in High- and Middle-Income Settings

#### Prevalence

- Malnutrition is not uncommon in children admitted to hospital, regardless of their underlying illness, and is often neglected in their specialist management.
- The prevalence of infants with FG admitted to hospital with illness-related and non-illness related etiology is not well known because of a lack of dynamic anthropometric measurements.
- Current studies report very different rates of disease-related malnutrition according to hospital type and size, methodology, and the population selected, with disease-related malnutrition prevalence ranging from 6% to 51% in hospitalized children.

#### Consequences

- Critically ill children who are malnourished on hospital admission have been shown to have increased risk of infection, duration of mechanical ventilation, length of stay, and increased mortality.
- Studies on mixed populations of hospitalized children have shown that malnutrition is associated with an increase in infectious complications and an increased length of stay.
- In the longer term malnourished children also have increased rates of impaired cognitive function and behavioral problems, including impaired communication skills and attention-deficit hyperactivity disorders.

#### Actions

- Clinicians should be alert to the risk of malnutrition in children admitted to hospital and identify those at risk; nutritional risk screening tools have been developed to help identify children at risk for malnutrition.
- Nutritional management of FG should consider the cause of the growth faltering and interventions should be tailored towards the underlying problem.
- Nutritional support given to critically ill malnourished children should take into account the phase of disease (acute, stable, and recovery).

## NON-DISEASE-RELATED FG

Based on the limited data available for countries that are classified as middle or high income we have extrapolated to estimate that the prevalence of children with sustained non-disease-related FG is <6% (47,48). It is important to acknowledge that most of the data on FG is observational and is largely from low-income settings. Not all studies use the definition of a fall in weight *z* score of ≥1.0 for growth faltering and there is generally a lack of differentiation between disease-related and non-disease-related growth faltering.

Most growth faltering in children in high- and middle-income settings is non-disease-related and organic disease is rare in otherwise well, asymptomatic children. It is most commonly caused by undernutrition relative to requirements and is usually related to a feeding problem, particularly difficulties in breast feeding, or short-lived illness (49). Infants are often over-investigated for an organic cause for growth faltering (49) and few investigations other than screening for urinary tract infection and celiac disease are recommended in an otherwise well child who is asymptomatic (8). The causes of non-disease-related FG in middle and high-income settings can be related to psychosocial, socioeconomic, and environmental factors including neglect and deprivation that impact on feeding practices (8,50,51). Preterm birth, neurodevelopmental concerns, and maternal depression and anxiety are also associated with non-organic FG (8). However, some children may have growth faltering attributed to a feeding problem when there is an undiagnosed medical condition, such as reflux.

The consequences of non-disease-related FG in middle- and high-income settings are similar to those in low-income settings and include impaired growth, cognition, behavior, and socioeconomic outcomes (25,52). Consequently, correcting for non-disease-related FG in childhood may improve cognitive achievements, school performance, and height growth (53,54).

### Agreed Statements: Non-Disease-Related FG in High- and Middle-Income Settings

#### Prevalence

From the limited data <6% of children in middle- and high-income settings have sustained non-disease-related FG, but often the overlap with disease-related FG is not considered.

#### Causes

The causes of non-disease-related FG in middle and high-income settings can be related to psychosocial, socioeconomic, and environmental factors.

#### Consequences

The consequences of FG may include an impact on schooling and cognitive achievements, short stature, and socioeconomic outcomes.

#### Benefits of Correcting

Correcting non-disease-related FG in childhood may improve cognitive achievements, school performance, and height growth.

## FG IN LOW-INCOME SETTINGS

Most FG leading to stunting occurs in South Asia, Africa, and Latin America; poor populations in rural areas are generally at greatest risk. Globally, 149 million children under 5 years were stunted according to 2020 figures (55), with the great majority living in Asia (53%) and Africa (41%). Child malnutrition is declining worldwide but only 25% of countries are on target to reach Sustainable Development Goals (SDGs) for 2030 (55). Infants born to short mothers are more likely to be stunted and, despite catch-up, babies short at birth are likely to remain shorter than those not short at birth. Wasted (defined as low weight for height) children are more likely to progress to become stunted.

It is difficult to ascertain causality between FG and later adverse outcomes because of potential confounding factors. Nonetheless, FG in LMICs commonly occurs together with numerous health and social outcomes, including poor brain development and delayed cognitive performance; delayed attainment of milestones; greater susceptibility to some infections; higher overall and disease-specific mortality in childhood; lower physical work capacity in adulthood; poorer earnings; and diminished human capital (56). Nearly half (43%) of deaths in children under 5 years of age are related to malnutrition (55).

FG may indicate the presence of an adverse health condition (such as infection(s) or an undetected congenital defect) and/or an inadequate diet or feeding regimen, which may be related to local custom in some low-income countries. Identifying and correcting the cause(s) of FG will greatly benefit the child in both the short- and the long-term. For most children with FG the cause is nutritional, however in low-income countries it commonly occurs together with gastroenteropathy and/or other pathology. Further investigation of a possible underlying medical cause for FG is generally indicated only where children have additional clear symptoms, for example vomiting, cough, or persistent diarrhea, or there are other concerns about possible underlying medical problems.

At the macro level economic development and reduction in socioeconomic inequalities is clearly the most effective mechanism to combat FG worldwide (57). However, economic development brings converse risks of obesity that must be addressed (55). At the community level mono-modal interventions show low efficacy and emphasize the need for combined approaches using nutrition-specific and nutrition-sensitive (addressing related concerns such as poor environmental hygiene) interventions (58).

FG has very early origins reflecting intergenerational constraints, poor fetal growth, and postnatal faltering initiated in early infancy (especially in the period 2–4 months postpartum) (59,60). Stunting results from recurrent episodes of weight faltering. Incident wasting at birth and in the first half of infancy has the greatest long-term impact (59). Interventions to combat FG are important at all ages but early interventions are most critical and it is important to try to focus on intervening at very early ages (58).

All nutritional interventions in LMICs should aim to normalize growth by eliminating, as far as possible, persistent FG. Interventions should not seek to promote growth in non-faltering children. Targeted interventions might therefore be required to avoid promotion of excess fat gain. Promotion of optimal breastfeeding practices should be the foundation of all early interventions (61). Subsequent interventions should focus on complementary feeding and optimizing toddler nutrition. These should seek to offer sufficient amounts of age-appropriate nutrient-dense foods, following WHO guidance on Infant and Young Child Feeding (IYCF) (61). Interventions in pregnancy are outside the scope of this review but over-zealous interventions in pregnancy might elicit adverse outcomes in the offspring; therefore, a counsel of patience is warranted. It may take several generations to safely rectify historical short stature in historically deprived populations (62).

In terms of future research needed in this field, some of the underlying causes of FG (eg, adverse gut microbiome) remain poorly understood and merit further investigation. Nutrition of both parents at the time of conception appear crucial to programming their offspring. This topic requires further research with a strong potential for guiding next-generation interventions.

### Agreed Statements: FG in Low-Income Settings

#### Prevalence

- Globally 149 million children under 5 years were stunted according to 2020 figures (54), with most living in Asia (53%) and Africa (41%).
- Child malnutrition is declining worldwide but only 25% of countries are on target to reach SDGs for 2030.

#### Causes

- For most children with FG the primary cause is nutritional.
- Further investigation of a possible underlying medical cause for FG is generally indicated only where children have additional symptoms, for example vomiting or cough or persistent diarrhea, or there are other concerns about possible underlying medical problems.

#### Consequences

- FG in LMICs commonly occurs together with numerous health and social outcomes, including poor brain development and delayed cognitive performance; delayed attainment of milestones; greater susceptibility to some infections; higher overall and disease-specific mortality in childhood; lower physical work capacity in adulthood; poorer earnings; and diminished human capital.

#### Management

- Identifying and correcting the cause(s) of FG will greatly benefit the child in both the short- and the long-term.
- At the macro level economic development and reduction in socioeconomic inequalities is the most effective mechanism to combat FG worldwide.
- At the community level mono-modal interventions show low efficacy; combined approaches are recommended using nutrition-specific and nutrition-sensitive interventions.
- All nutritional interventions in LMICs should aim to normalize growth by eliminating, as far as possible, persistent FG.
- Promotion of optimal breastfeeding practices should be the foundation of all early interventions.
- Interventions should seek to offer sufficient amounts of age-appropriate nutrient-dense foods, following WHO guidance on IYCF.

## NUTRITIONAL MANAGEMENT OF DISEASE-RELATED AND NON-DISEASE-RELATED FG

**FIGURE 2.**
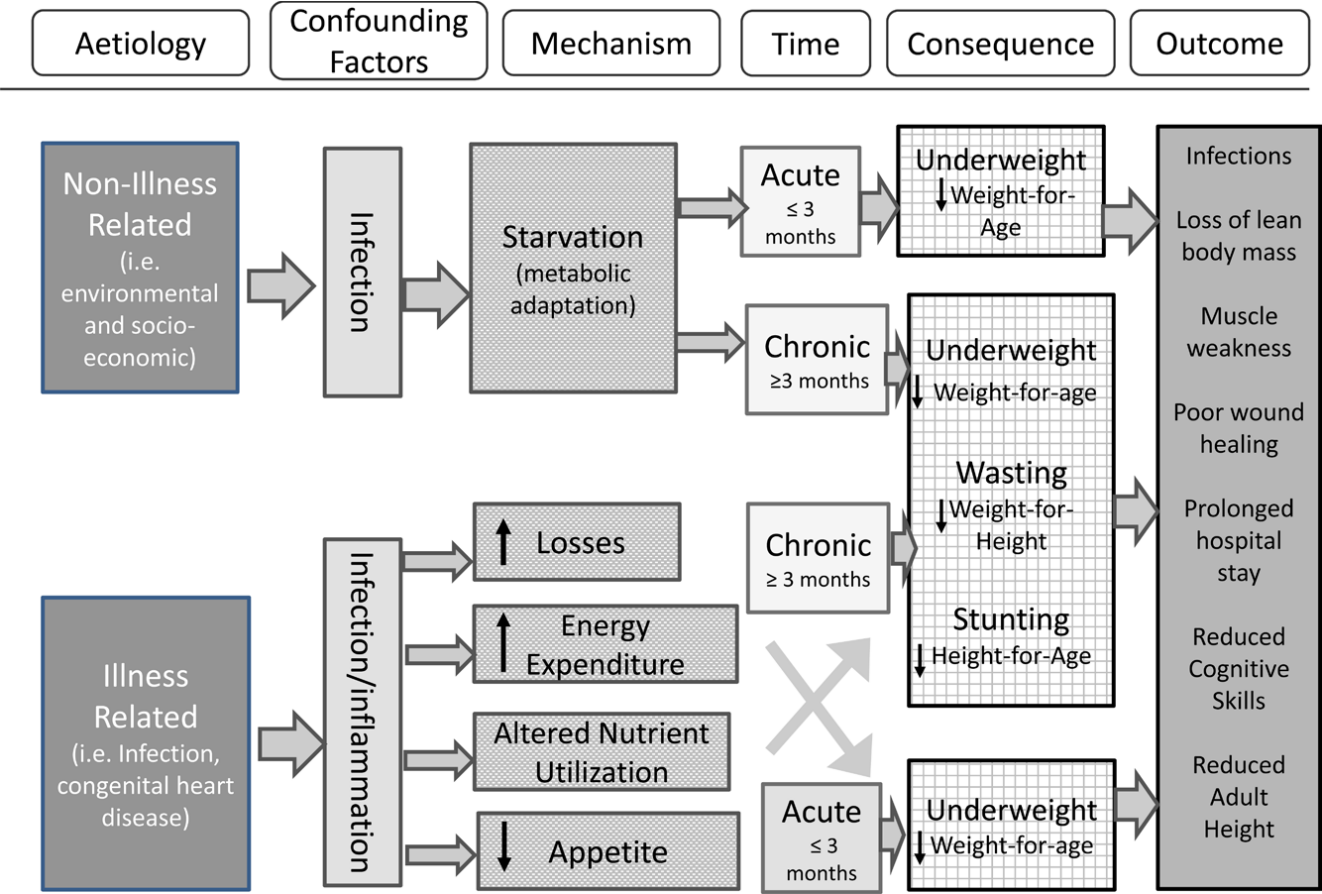
Algorithm summarizing the factors to consider when planning interventions to manage faltering growth. Adapted from Meyer and Marino (78).

The nutritional management for infants and young children from low-, middle-, and high-income countries should consider the cause of the growth faltering and interventions toward addressing the underlying problem (25) (Figure 2).

Nutritional management of disease- and non-disease-related FG requires both macro- and micronutrients for optimal catch-up (63,64). In disease-related FG these requirements are, where data or guidelines are available, specific to the underlying diagnosis, with specific characteristics that influence intake, digestion, and losses, and it is important to take these into account when developing the dietary plan (65). Initiation of nutritional support for infants with disease-related FG should consider the phase of illness (acute, stable, and recovery phase) (Figure 2) and avoid overfeeding in the acute phase of disease (40). Nutritional management of non-disease-related FG should also manage the underlying cause, which, very broadly, may include feeding difficulties, inappropriate dietary elimination, and food security (25,50,66).

Breastfeeding should be supported in both disease and non-disease-related FG by assessing technique and supply as a first priority, and only where appropriate and if of significant severity, infant milk fortification, cup feeding, or supplementary formula should be considered (67). In formula fed infants, ready to use energy dense therapeutic feeds with proven efficacy should be used, where available (65,68). In the many countries where these are not available suitable locally available powdered feeds can be used, applying WHO hygiene safety for mixing (68).

It is very important that modular additions of only fat and carbohydrates to feed and food should be avoided, as this reduces the protein:energy ratio (63). When only carbohydrates and/or fat are added to infant feeds the energy percentage from protein is significantly reduced, which has been shown to impact on the ratio of lean to FM in catch-up growth. The addition of carbohydrates increases the osmolality of feeds, which may affect gastric emptying kinetics with a potentially negative impact in infants that are already medically fragile (44,69).

In infants with FG, where complementary feeding has been started, the fortification of accepted foods and advice on foods that are naturally energy dense, locally available, and culturally acceptable form an essential part of the management (8,70,71). In cases where the nutritional requirements of the nutritional management plan cannot be met through oral intake, the plan should include recommendations for enteral (ie, tube) feeding, including monitoring by a multidisciplinary nutrition support team to minimize the risk of enteral nutrition (EN)-associated complications (72). In addition, micronutrient deficiencies should be corrected as part of the nutritional treatment plan, with appropriate therapeutic dosages and monitoring of the intervention at an appropriate interval for the deficiency (73,74).

Monitoring of a nutritional management plan should always include a target for appropriate catch-up growth that is monitored at an interval that is considered appropriate by the health care professional and the available health care service for the severity of growth faltering (64). We cannot set generalized prescriptive targets because these depend on the severity and causes of FG, but it is essential to set a target and monitor appropriately. However, we suggest that a pragmatic target for catch-up growth should return the child to his/her original WFA *z* score before experiencing growth faltering. Importantly, misplaced fear of promoting accelerated (too rapid) growth is often due to rapid gains in weight that are not accompanied by catch-up growth in length.

### Agreed Statements

- Nutritional management of FG should consider the cause of the growth faltering and intervention should be tailored towards the underlying problem (25).
- Nutritional management of disease- and non-disease-related FG requires a balanced ratio of energy and protein in addition to micronutrients for optimal catch-up (64,75).
- Nutritional management of disease-related FG should consider macro and micronutrient requirements specific to the underlying diagnosis (76).
- Nutritional management of non-disease-related FG should also manage the underlying cause which may include feeding difficulties, inappropriate dietary elimination, and food security (25,50,66).
- Breastfeeding should be supported in both disease- and non-disease-related FG by ensuring assessing technique and supply and only where appropriate infant milk fortification, cup feeding, or supplementary formula should be considered (67).
- In formula-fed infants ready to use energy dense therapeutic feeds with proven efficacy should be used, where available; if these are not available suitable locally available powdered feeds can be used, applying WHO hygiene safety for mixing (65,68).
- Modular additions of only fat and carbohydrates to feed and food should be avoided, as this reduces the protein energy ratio (75).
- Nutritional management for both medical and non-medical FG should include either/both the fortification of accepted foods and advice on foods that are naturally energy dense and locally available (8,70,71). In the case when nutritional requirements of the nutritional management plan cannot be met through oral intake, a nutritional management plan should entail enteral (ie, tube) feeding. Monitoring by a multidisciplinary nutrition support team should be provided to minimize the risk of EN-associated complications (72). In LMICs children with acute severe malnutrition who fail the appetite test and/or are clinically unwell should be treated as inpatients. WHO guidelines should be followed to determine the need for vitamin A supplementation or antibiotic treatment (77).
- Micronutrient deficiencies need to be corrected as part of the nutritional treatment plan, with appropriate therapeutic dosages and monitoring of the intervention is required at an interval appropriate for the deficiency (73,74).
- The nutritional management plan should include a target for appropriate catch-up growth that is monitored at an interval that is deemed appropriate by the health care professional, the available health care service, and the severity of the FG (64).

## FUTURE RESEARCH NEEDS

- Some of the underlying causes of FG (eg, adverse gut microbiome) remain poorly understood and merit further investigation.
- Research is required to identify cost-effective and implementable community interventions to combat growth faltering in under-privileged populations.
- Research is needed on the alterations of body composition concerning FM and FFM after catch-up growth in the short term and the metabolic consequences in the long term.
- Properly designed randomized controlled trials should be initiated to further clarify optimal intervention strategies for FG and the focus should be on both weight and height.
- Research is needed focusing on the causes and management of faltering in linear growth. More experimental research is needed to establish causality for the causes, management, and consequences of both growth faltering and growth acceleration.

## CONCLUSIONS

Faltering growth in infants and children <2 years of age is a common problem regularly seen by health care professionals. It has multiple causes and occurs due to disease-related and non-disease related factors, and in low-, middle-, and high- income settings. However, in all settings it is essential to detect FG, address underlying causes and support catch-up growth where this is indicated.

## References

[1] McCance RA Widdowson EM. The determinants of growth and form. Proc R Soc Lond B Biol Sci. 1974;185:1–17. 4149051 10.1098/rspb.1974.0001

[2] Widdowson EM. Cellular growth and function. Proc Nutr Soc. 1976;35:357–62. 800659 10.1079/pns19760056

[3] Barker D. Fetal and Infant Origins of Adult Disease. London, UK: British Medical Journal; 1992.

[4] Barker DJP. The origins of the developmental origins theory. J Intern Med. 2007;261:412–7. 17444880 10.1111/j.1365-2796.2007.01809.x

[5] Tanner JM. Growth as a mirror of the condition of society: secular trends and class distinctions. Demirjan A, ed. In: Human Growth: A Multidisciplinary Review. London: Taylor and Francis; 1986:3–34.

[6] World Health Organization. WHO Global Database on Child Growth and Malnutrition. Geneva: World Health Organization/Department of Nutrition for Health and Development CH – 1211; 2014;27.

[7] Hermanussen M. Basics. Hermanussen M, ed. In: Auxology Studying Human Growth and Development. Stuttgart: Schweizerbart’sche; 2013.

[8] National Institute for Health and Care Excellence. Faltering growth: recognition and management of faltering growth in children. NICE guideline [NG75]. Published: 27 September 2017. Available at: www.nice.org.uk/guidance/ng75. Accessed September 26, 2021. 28991420

[9] Prader A Tanner JM von Harnack G. Catch-up growth following illness or starvation. An example of developmental canalization in man. J Pediatr. 1963;62:646–59. 13985887 10.1016/s0022-3476(63)80035-9

[10] Tanner JM. Catch-up growth in man. Br Med Bull. 1981;37:233–8. 7034846 10.1093/oxfordjournals.bmb.a071708

[11] de Wit C Sas TCJ Wit JM Cutfield WS. Patterns of catch-up growth. J Pediatr. 2012;162:415–20. 23153864 10.1016/j.jpeds.2012.10.014

[12] Sinha RK Kumar P Daniel A. Association between anthropometric criteria and body composition among children aged 6–59 months with severe acute malnutrition: a cross-sectional assessment from India. BMC Nutr. 2022;8:56. 35739560 10.1186/s40795-022-00551-6 PMC9219132

[13] Slaughter MH Lohman TG Boileau RA. Skinfold equations for estimation of body fatness in children and youth. Hum Biol. 1988;60:709–23. 3224965

[14] Aris IM Soh SE Tint MT. Body fat in Singaporean infants: development of body fat prediction equations in Asian newborns. Eur J Clin Nutr. 2013;67:922–7. 23549200 10.1038/ejcn.2013.69

[15] Catalano PM Thomas AJ Avallone DA Amini SB. Anthropometric estimation of neonatal body composition. Am J Obstet Gynecol. 1995;173:1176–81. 7485315 10.1016/0002-9378(95)91348-3

[16] van Beijsterveldt IALP Snowden SG Myers PN. Metabolomics in early life and association with body composition at age 2 years. Pediatr Obes. 2022;3:e12859. 10.1111/ijpo.12859 PMC9286420 34644810

[17] Singhal A Lucas A. Early origins of cardiovascular disease: is there a unifying hypothesis? Lancet. 2004;363:1642–5. 15145640 10.1016/S0140-6736(04)16210-7

[18] Singhal A. The role of infant nutrition in the global epidemic of non-communicable disease. Proc Nutr Soc. 2016;75:162–8. 27066843 10.1017/S0029665116000057

[19] Woo Baidal JA Locks LM Cheng ER Blake-Lamb TL Perkins ME Taveras EM. Risk factors for childhood obesity in the first 1,000 days; a systematic review. Am J Prev Med. 2016;50:761–79. 26916261 10.1016/j.amepre.2015.11.012

[20] Druet C Stettler N Sharp S. Prediction of childhood obesity by infancy weight gain: an individual-level meta-analysis. Paediatr Perinat Epidemiol. 2012;26:19–26. 22150704 10.1111/j.1365-3016.2011.01213.x

[21] Pesch MH Levitt KJ Danziger P Orringer K. Pediatrician’s beliefs and practices around rapid infant weight gain: a qualitative study. Glob Pediatr Health. 2021;8:1–10. doi:10.1177/2333794X21992164. 10.1177/2333794X21992164 PMC7874340 33614855

[22] Elke G Wang M Weiler N Day AG Heyland DK. Close to recommended caloric and protein intake by enteral nutrition is associated with better clinical outcome of critically ill septic patients: secondary analysis of a large international nutrition database. Crit Care. 2014;18:R29. 24506888 10.1186/cc13720 PMC4056527

[23] Mehta NM Skillman HE Irving SY. Guidelines for the provision and assessment of nutrition support therapy in the pediatric critically Ill patient: Society of Critical Care Medicine and American Society for Parenteral and Enteral Nutrition. Pediatr Crit Care Med. 2017;18:675–715. 28691958 10.1097/PCC.0000000000001134

[24] Pollack MM Wilkinson JD Glass NL. Long-stay pediatric intensive care unit patients: outcome and resource utilization. Pediatrics. 1987;80:855–60. 3684396

[25] Mehta NM Corkins MR Lyman B. Defining pediatric malnutrition: a paradigm shift toward etiology-related definitions. JPEN J Parenter Enteral Nutr. 2013;37:460–81. 23528324 10.1177/0148607113479972

[26] Goulet O. Assessment of nutritional status in clinical practice. Baillieres Clin Gastroenterol. 1998;12:647–69. 10079901 10.1016/s0950-3528(98)90002-1

[27] Hulst J Joosten K Zimmerman L. Malnutrition in critically ill children: from admission to 6 months after discharge. Clin Nutr. 2004;23:223–32. 15030962 10.1016/S0261-5614(03)00130-4

[28] Becker PJ Bellini SG Vega MW. Validity and reliability of pediatric nutrition screening tools for hospital, outpatient, and community settings: a 2018 evidence analysis center systematic review. J Acad Nutr Diet. 2020;120:288–318. 31547992 10.1016/j.jand.2019.06.257

[29] Klanjsek P Panjnkihar M Varda NM Brzan PP. Screening and assessment tools for early detection of malnutrition in hospitalised children: a systematic review of validation studies. BMJ Open. 2019;9:e025444. 10.1136/bmjopen-2018-025444 PMC6549612 31138579

[30] Huysentruyt K Vandenplas Y De Schepper J. Screening and assessment tools for pediatric malnutrition. Curr Opin Clin Nutr Metab Care. 2016;19:336–40. 27327411 10.1097/MCO.0000000000000297

[31] de Menezes FS Leite HP Koch Nogueira PC. Malnutrition as an independent predictor of clinical outcome in critically ill children. Nutrition. 2012;28:267–70. 21872433 10.1016/j.nut.2011.05.015

[32] Bagri NK Jose B Shah SK Bhutia TD Kabra SK Lodha R. Impact of malnutrition on the outcome of critically Ill children. Indian J Pediatr. 2015;82:601–5. 25804317 10.1007/s12098-015-1738-y

[33] Drotar D Sturm L. Prediction of intellectual development in young children with early histories of nonorganic failure-to-thrive. J Pediatr Psychol. 1988;13:281–96. 3171820 10.1093/jpepsy/13.2.281

[34] Black MM Krishnakumar A. Predicting longitudinal growth curves of height and weight using ecological factors for children with and without early growth deficiency. J Nutr. 1999;129:539S–43S. 10064327 10.1093/jn/129.2.539S

[35] Drewett RF Corbett SS Wright CM. Cognitive and educational attainments at school age of children who failed to thrive in infancy: a population-based study. J Child Psychol Psychiatry. 1999;40:551–61. 10357162

[36] Rudolf MC Logan S. What is the long term outcome for children who fail to thrive? A systematic review. Arch Dis Child. 2005;90:925–31. 15890695 10.1136/adc.2004.050179 PMC1720590

[37] Corbett SS Drewett RF. To what extent is failure to thrive in infancy associated with poorer cognitive development? A review and meta-analysis. J Child Psychol Psychiatry. 2004;45:641–54. 15055382 10.1111/j.1469-7610.2004.00253.x

[38] Galler JR Ramsey F Solimano G Lowell WE Mason E. The influence of early malnutrition on subsequent behavioral development, I: degree of impairment in intellectual performance. J Am Acad Child Psychiatry. 1983;22:8–15. 6402542 10.1097/00004583-198301000-00002

[39] Black MM Dubowitz H Krishnakumar A Starr RH Jr. Early intervention and recovery among children with failure to thrive: follow-up at age 8. Pediatrics. 2007;120:59–69. 17606562 10.1542/peds.2006-1657

[40] Joosten KF Kerklaan D Verbruggen SC. Nutritional support and the role of the stress response in critically ill children. Curr Opin Clin Nutr Metab Care. 2016;19:226–33. 26963579 10.1097/MCO.0000000000000268

[41] de Menezes FS Leite HP Fernandez J Benzecry SG de Carvalho WB. Hypophosphatemia in critically ill children. Rev Hosp Clin Fac Med Sao Paulo. 2004;59:306–11. 15543405 10.1590/s0041-87812004000500015

[42] Byrnes MC Stangenes J. Refeeding in the ICU: an adult and pediatric problem. Curr Opin Clin Nutr Metab Care. 2011;14:186–92. 21102317 10.1097/MCO.0b013e328341ed93

[43] Pulcini CD Zettle S Srinath A. Refeeding syndrome. Pediatr Rev. 2016;37:516–23. 27909106 10.1542/pir.2015-0152

[44] Clarke SE Evans S Macdonald A Davies P Booth IW. Randomized comparison of a nutrient-dense formula with an energy-supplemented formula for infants with faltering growth. J Hum Nutr Diet. 2007;20:329–39. 17635310 10.1111/j.1365-277X.2007.00805.x

[45] Smith C McCabe H Macdonald S. Improved growth, tolerance and intake with an extensively hydrolysed peptide feed in infants with complex disease. Clin Nutr. 2018;37:1005–12. 28506449 10.1016/j.clnu.2017.04.012

[46] Eveleens RD Dungen DK Verbruggen SCAT Hulst JM Joosten KFM. Weight improvement with the use of protein and energy enriched nutritional formula in infants with a prolonged PICU stay. J Hum Nutr Diet. 2019;32:3–10. 30318663 10.1111/jhn.12603

[47] Wright CM Garcian AL. Child undernutrition in affluent societies: what are we talking about? Proc Nutr Soc. 2012;71:545–55. 22954067 10.1017/S0029665112000687

[48] McAlpine J Nielsen DK Lee J Larsen BMK. Growth faltering: the new and the old. Clin Pediatr. 2019;2:1012.

[49] Shields B Wacogne I Wright CM. Weight faltering and failure to thrive in infancy and early childhood. BMJ. 2012;345:e5931. 23014901 10.1136/bmj.e5931

[50] Wright CM Parkinson KN Drewett RF. The influence of maternal socio-economic and emotional factors on infant weight gain and weight faltering (failure to thrive): data from a prospective birth cohort. Arch Dis Child. 2006;91:312–7. 16397011 10.1136/adc.2005.077750 PMC2065961

[51] Malone C Sharif F Glennon-Slattery C. Growth and nutritional risk in children with developmental delay. Ir J Med Sci. 2016;185:839–46. 26573207 10.1007/s11845-015-1377-3

[52] Menezes AMB Oliveira PD Wehrmeister FC. Associations between growth from birth to 18 years, intelligence, and schooling in a Brazilian cohort. Am J Clin Nutr. 2020;112:187–94. 32239193 10.1093/ajcn/nqaa047 PMC7326584

[53] Lundeen EA Behrman JR Crookston BT. Growth faltering and recovery in children aged 1–8 years in four low- and middle-income countries: young Lives. Public Health Nutr. 2014;17:2131–7. 24477079 10.1017/S1368980013003017 PMC4043952

[54] Crookston BT Schott W Cueto S. Postinfancy growth, schooling, and cognitive achievement: young Lives. Am J Clin Nutr. 2013;98:1555–63. 24067665 10.3945/ajcn.113.067561 PMC3831540

[55] World Health Organization. UNICEF/WHO/The World Bank Group joint child malnutrition estimates: key findings of the 2021 edition. 2021. Available at: https://www.who.int/publications/i/item/9789240025257. Accessed July 30, 2022.

[56] World Food Programme. 2017 – Working for zero hunger. 2017. Available at: https://www.wfp.org/publications/2017-working-zero-hunger. Accessed July 30, 2022.

[57] Victora CG Christian P Vidaletti LP. Revisiting maternal and child undernutrition in low-income and middle-income countries: variable progress towards an unfinished agenda. Lancet. 2021;397:1388–99. 33691094 10.1016/S0140-6736(21)00394-9 PMC7613170

[58] Bhutta ZA Das JK Rizvi A. Evidence-based interventions for improvement of maternal and child nutrition: what can be done and at what cost? Lancet. 2013;382:452–77. 23746776 10.1016/S0140-6736(13)60996-4

[59] Mertens A Benjamin-Chung J Colford JM Jr. Causes and consequences of child growth failure in low- and middle-income countries. medRxiv. 2020. doi:10.1101/2020.06.09.20127100.

[60] Local Burden of Disease Child Growth Failure Collaborators. Mapping child growth failure across low- and middle-income countries. Nature. 2020;577:231–4. 31915393 10.1038/s41586-019-1878-8 PMC7015855

[61] WHO/UNICEF. Global Strategy for Infant and Young Child Feeding. WHO; 2003. Available at: https://www.who.int/publications/i/item/9241562218. 15806879

[62] Hardikar AA Satoor SN Karandikar MS. Multigenerational undernutrition increases susceptibility to obesity and diabetes that is not reversed after dietary recuperation. Cell Metab. 2015;22:312–9. 26166746 10.1016/j.cmet.2015.06.008

[63] World Health Organization. Report of a Joint WHO/FAO/UNU Expert Consultation. Protein and Amino Acid Requirements in Human Nutrition. 2007. WHO Technical Report Series. Report 935. Available at: https://apps.who.int/iris/bitstream/handle/10665/43411/WHO_TRS_935_eng.pdf?sequence=1&isAllowed=y. Accessed September 26, 2021. 18330140

[64] Golden MH. Proposed recommended nutrient densities for moderately malnourished children. Food Nutr Bull. 2009;30:S267–342. 19998863 10.1177/15648265090303S302

[65] Marino LV Meyer R Cooke ML. Cost comparison between powdered versus energy dense infant formula for undernourished children in a hospital setting. e-SPEN J. 2013;8:e145–9.

[66] Mank I Vandormael A Traoré I. Dietary habits associated with growth development of children aged < 5 years in the Nouna Health and Demographic Surveillance System, Burkina Faso. Nutr J. 2020;19:81. doi:10.1186/s12937-020-00591-3. 32772913 10.1186/s12937-020-00591-3 PMC7416397

[67] Rana R McGrath M Gupta P Thakur K Kerac K. Feeding interventions for infants with growth failure in the first six months of life: a systematic review. Nutrients. 2020;12:2044. doi:10.3390/nu12072044. 32660020 10.3390/nu12072044 PMC7400880

[68] World Health Organization. Severe Malnutrition: Report of a Consultation to Review Current Literature. 2005. Available at: https://apps.who.int/iris/bitstream/handle/10665/43253/9241593318-eng.pdf. Accessed September 26, 2021.

[69] Romera G Figueras J Rodríguez-Miguélez JM Ortega J Jiménez R. Energy intake, metabolic balance and growth in preterm infants fed formulas with different nonprotein energy supplements. J Pediatr Gastroenterol Nutr. 2004;38:407–13. 15085019 10.1097/00005176-200404000-00008

[70] Okeyo DO. Impact of food fortification on child growth and development during complementary feeding. Ann Nutr Metab. 2018;73:7–13. 10.1159/000490087 30196295

[71] Gonzelez-Viana E Dworzynski K Murphy MS Peek R. Faltering growth in children: summary of NICE guidance. BMJ. 2017;358:j4219. doi:10.1136/bmj.j4219. 28963099 10.1136/bmj.j4219

[72] Braegger C Decsi T Dias JA. Practical approach to paediatric enteral nutrition: a comment by the ESPGHAN Committee on Nutrition. J Pediatr Gastroenterol Nutr. 2010;51:110–22. 20453670 10.1097/MPG.0b013e3181d336d2

[73] World Health Organization. Health Topics. Micronutrients. 2021. Available at: https://www.who.int/health-topics/micronutrients#tab=tab_1. Accessed September 26, 2021.

[74] Gerasimidis K Bronsky J Catchpole A. Assessment and interpretation of vitamin and trace element status in sick children: a position paper from the European Society for Paediatric Gatroenterology Hepatology, and Nutrition Committee on Nutrition. ESGPHAN paper. J Pediatr Gastroenterol Nutr. 2020;70:873–81. doi:10.1097/MPG.0000000000002688. 32443051 10.1097/MPG.0000000000002688

[75] World Health Organization. Joint FAO/WHO/UNU Expert Consultation on Protein and Amino Acid Requirements in Human Nutrition (2002: Geneva, Switzerland), Food and Agriculture Organization of the United Nations, World Health Organization & United Nations University. 2007. Protein and amino acid requirements in human nutrition: report of a joint FAO/WHO/UNU expert consultation. Available at: https://apps.who.int/iris/handle/10665/43411. Accessed September 26, 2021.

[76] Marino L. Growth faltering in children: manual of dietetics. Gandy J, ed. In: Manual of Dietetics. 2019. Available at: https://doi.org/978-1119235927. Accessed September 26, 2021.

[77] World Health Organization. Guideline: Updates on the Management of Severe Acute Malnutrition in Infants and Children. 2013. Available at: https://www.who.int/publications/i/item/9789241506328. Accessed September 26, 2021. 24649519

[78] Meyer R Marino L. Nutrition support in paediatrics. Hickson M Smith S Whelan K, eds. In: Advanced Nutrition and Dietetics in Nutrition Support. 2018. Available at: 10.1002/9781118993880.ch5.1. Accessed September 26, 2021.

